# HIV Cerebrospinal Fluid Escape: Interventions for the Management, Current Evidence and Future Perspectives

**DOI:** 10.3390/tropicalmed10020045

**Published:** 2025-02-05

**Authors:** Sophie H. Kelly, Sam Nightingale, Ravindra K. Gupta, Dami A. Collier

**Affiliations:** 1 Cambridge University Hospitals NHS Foundation Trust, Cambridge CB2 0QQ, UK; rkg20@cam.ac.uk; 2 Department of Medicine, University of Cambridge, Cambridge CB2 1TN, UK; 3 Neuroscience Institute, University of Cape Town, Cape Town 7700, South Africa; sam.nightingale@uct.ac.za; 4 Cambridge Institute of Therapeutic Immunology & Infectious Disease (CITIID), Cambridge CB2 0AW, UK; 5 Africa Health Research Institute, Durban 4013, South Africa; 6 Department of Pathology, University of Cambridge, Cambridge CB2 1TN, UK

**Keywords:** HIV, cerebrospinal fluid, CSF, compartmentalization, escape

## Abstract

Neurocognitive impairment is an important cause of HIV-associated morbidity. The advent of antiretroviral therapy (ART) has shifted the spectrum of HIV-associated cognitive impairment from HIV-associated dementia to milder forms of cognitive impairment. Independent replication of HIV within the central nervous system in those on effective ART with peripheral suppression is a recognised phenomenon known as cerebrospinal fluid (CSF) HIV RNA escape. CSF HIV RNA escape is independently associated with neurocognitive impairment but has also been detected in asymptomatic persons with HIV. The current consensus for management of CSF HIV RNA escape is based on expert opinion rather than empirical evidence. The current evidence suggests having a low threshold to investigate for CSF HIV RNA escape and optimising ART based on resistance profiles. The use of central nervous system (CNS) penetration effectiveness scores is no longer recommended. The evidence for statins, SSRIs, minocycline, lithium and valproate is limited to small-scale studies. There are potential new developments in the form of nanoparticles, Janus Kinase inhibitors and latency reversal agents.

## 1. Background

### 1.1. Introduction

Thirty-nine million people are living with human immunodeficiency virus (HIV) globally [1]. The impact of HIV on the brain was established as early as 1986 with the characterisation of the AIDS dementia complex [2,3,4]. The use of antiretroviral therapy (ART) markedly reduced the prevalence of severe cognitive impairment associated with HIV [5,6,7]. However, there is the persistence of mild cognitive impairment despite adequate treatment with ART [8]. As HIV treatment and outcomes have improved, there has been a shift in the classification of cognitive impairment in people with HIV away from the Frascati criteria for HIV-associated neurocognitive disorder (HAND) towards one that recognises the multifactorial nature of this condition and differentiates active or legacy HIV-associated brain injury (HABI) from other potential causes of cognitive impairment [9,10]. The pathophysiology of HABI is not fully understood. It is thought to occur via multiple mechanisms, including virus-induced neuronal damage, compromised blood–brain barrier (BBB) function and chronic immune activation [9]. Neuroinflammation can occur following viral entry and replication in the brain [11,12,13,14]. HIV CNS entry occurs in the acute phase of infection and is near universal [15]. In times of high viral load, either through direct viral toxicity or due to the subsequent inflammatory response, the tight junctions of the BBB are disrupted [16,17,18,19]. The virus can cross the BBB directly and through infected monocytes and T cells—this establishes a reservoir of infection in microglia, perivascular macrophages and astrocytes [20,21]. There is evidence that the BBB disruption that occurs in primary CNS infection can persist despite treatment with ART [22]. Once HIV has entered the CNS, it is exposed to a distinct microenvironment [23]. This allows for the development of compartmentalised infection and the evolution of genetically distinct quasispecies within the CNS compartment [24]. Viral populations have been reported to sometimes evolve from R5 T cell-tropic virus to R5 macrophage-tropic virus with a greater ability to infect myeloid-derived cells in the CNS [24,25,26,27,28]. Productive infection is predominantly found in perivascular macrophages and microglia. Infection is associated with inflammation and immune activation of glial cells, which contributes to neuronal injury [13]. Furthermore, some viral proteins, for example Tat and gp120, can directly cause neuronal injury [29].

CSF HIV RNA escape is defined as CSF HIV RNA concentration that is higher than plasma HIV RNA concentration [30]. The reported prevalence of CSF HIV RNA escape varies between studies, with estimates ranging from 1 to 28.6% of PLWH on ART [31,32,33,34,35,36,37,38,39,40,41,42,43]. However, some of this variation may relate to the population studied, indication for LP and differences in the definitions of CSF HIV RNA escape used. No distinctive clinical characteristics can predict CSF HIV RNA escape, and no biomarkers are in clinical use [43]. CSF HIV RNA escape is a known cause of HABI [44,45].

### 1.2. Aims of Review

There are few consensus guidelines on the treatment of CSF HIV RNA escape. The British HIV Association (BHIVA) and European Aids Clinical Society (EACS) recommend sampling CSF in those with cognitive impairment and an undetectable viral load in the absence of another cause and, if there is a detectable CSF viral load, genotyping this virus for drug resistance mutations [46,47]. The BHIVA and EACS recommend avoiding two drug regimens, protease inhibitors and raltegravir, and suggest including a dual nucleoside backbone with consideration of an increased dose of dolutegravir [46,47]. Over recent years, there has been growing interest in repurposing already licensed drugs for the treatment of HIV CNS disease and in developing newer technologies such as nanoparticles. This review summarises the currently available evidence for the treatment of CSF HIV RNA escape and directions for future research.

## 2. Methods and Definitions

### 2.1. Methods

Databases including PubMed, Medline, Embase and Cochrane were searched for the following terms: “HIV” or “Human Immunodeficiency Virus” or “Acquired Immunodeficiency Syndrome” or “AIDS” and “Cerebrospinal fluid” or “CSF” and “escape” or “discordance” or “discordant”; this was performed for texts published up to October 2024. Inclusion criteria were texts available in the English language that included CSF viral load before and after initiation of an intervention or treatment; exclusion criteria were texts that only reported cognitive outcomes. We included all study types, case reports and case series. We assessed the strength of evidence based on the modified the (GRADE) system adopted by BHIVA [46,48]; we report recommendations as described in current international guidelines.

### 2.2. Definition of CSF HIV RNA Escape

The consensus on the definition of CSF HIV RNA escape has been most recently agreed upon at the Second Global HIV Cerebrospinal Fluid Escape Consortium in 2019 [30]. At this meeting, three key recommendations were made: (i) CSF HIV RNA escape should be defined as any detectable CSF RNA level where plasma RNA level is undetectable and any CSF HIV RNA level that is greater than the plasma HIV RNA level when plasma RNA is detectable; (ii) CSF HIV RNA escape should be classified as symptomatic or asymptomatic based on the reported symptoms of the patient rather than the indication for lumbar puncture; and (iii) CSF HIV RNA escape should be actively managed when the patient is symptomatic [30].

### 2.3. Types of Escape

Table 1 shows a schema for categorising CSF HIV RNA escape. Typically, three types of CSF HIV RNA escape have been described: symptomatic, asymptomatic and secondary [49]. “Symptomatic” CSF HIV RNA escape refers to primary CSF HIV RNA escape, that occurs in the absence of another CNS opportunistic infection, in people with neurological symptoms. “Asymptomatic” CSF HIV RNA escape refers to primary CSF HIV RNA escape in the absence of neurological symptoms. “Secondary” CSF HIV RNA escape refers to CSF HIV RNA escape that occurs in the context of another cause of CSF pleocytosis, such as a CNS opportunistic infection.

We have proposed a fourth category of artefactual CSF escape to describe the presence of HIV RNA at higher levels in CSF than plasma at levels within the variability of the HIV RNA assay used (correlation of variation in commonly used assays is 32% to 77% at low viral loads) and maybe related to differential suppression rates between the CSF and plasma compartments [50]. The inclusion of this category recognises that, within the current accepted classification system, some people who do not have true asymptomatic escape will be classified as having asymptomatic escape due to the variability of the assays. We believe that this is a conceptually distinct entity to true asymptomatic escape and should be recognised in the fourth category of “Artefactual” CSF HIV RNA escape.

### 2.4. Epidemiology and Clinical Context

CSF HIV RNA escape has a broad spectrum of clinical manifestations, ranging from asymptomatic to neurological symptoms such as cognitive impairment, headaches, sleep disturbance, seizures, ataxia, personality disorders, psychosis, coma and death [43,49,51,52,53,54].

The clinical significance of CSF HIV RNA escape has been reviewed in more detail by this group elsewhere [14]. Cognitive impairment in people with HIV is often multifactorial and can be caused by opportunistic infection, ART toxicity and other non-HIV related causes [8,55]. CSF HIV RNA escape has been associated with poor scores on neuropsychological testing in people with HIV, and HIV-1 RNA transcripts are associated with neuroinflammation and cognitive impairment [56]. CSF HIV RNA escape is not the most common cause of cognitive impairment in people with HIV; studies have found CSF HIV RNA escape to be present in between 10 and 20% of people presenting with neurological symptoms [55]. Asymptomatic escape is present in between 1 and 2% of people living with HIV on effective ART [57,58,59]. The significance of asymptomatic escape is not clear, and it is not always associated with HABI. The current consensus opinion on management is to treat those with neurological symptoms and to do so with ART intensification [9]. In Europe and the UK, testing for CSF HIV viral load is recommended in anyone presenting with cognitive impairment and viral suppression after other causes of the symptoms, including psychiatric causes and opportunistic infections, have been excluded [10,46]. This algorithmic protocol is not standardised worldwide and is limited by access and acceptability of CSF testing.

**Table 1:** A schema for categorising CSF HIV RNA escape.

	Typical Clinical Presentation	Prevalence and Relative Frequency	Typical CSF Findings	Typical Imaging Findings
**Symptomatic** Primary CSF HIV RNA escape presenting with neurological symptoms	Variable presentations can include: Cognitive slowing Headaches Sleep disturbance Seizures Ataxia Personality change Psychosis Reduced level of consciousness [14]	In two large cohort studies of people on ART, the achieved viral suppression overall prevalence was estimated at 0.09–2.5% [35,36] * 35.1–38.2% of aviraemic patients with CSF escape had neurocognitive impairment [35,36] **	Lymphocytic pleocytosis High neuroinflammatory markers High levels of neurofilament light chain [60,61] High levels of CSF neopterin	Diffuse white matter signal abnormalities on brain MRI [33]
**Asymptomatic** Primary CSF HIV RNA escape presenting without any neurological symptoms	No symptoms Can be transient (CSF blips)	In two large cohort studies of people on ART, the achieved viral suppression overall prevalence was estimated as 2.7–4.7% [35,36] 61.8–64.9% of aviraemic patients with CSF escape had no neurocognitive symptoms [35,36] ***	Can have mildly raised CSF white cell count [60,62] Lower CSF neurofilament light chain compared to symptomatic escape [60] Can have raised neopterin, though typically lower than symptomatic or secondary escape [32,60]	Not associated with changes in brain imaging
**Secondary** CSF HIV RNA escape in the presence of an alternative cause of CSF pleocytosis	May have symptoms in the context of another neurological infection or autoimmune condition	Limited evidence In one study, 33.8% of virally suppressed people with CSF escape had a neurological coinfection [35]	Related to the neurological infection High levels of CSF neopterin	Related to the underlying neurological infection
**Artefactual** CSF HIV RNA viral load higher than HIV RNA plasma viral load within the variability of the assay used	No symptoms	Unknown	No lymphocytic pleocytosis	Not associated with changes in brain imaging

* Mukerji et al.; cohort of 1063 people, the overall prevalence of CSF escape with symptoms reported was 35.1% (*n* = 27) of participants with CSF escape. The authors reported that 33.8% (*n* = 26) of patients with CSF escape have a neurological co-infection, but it is not clear how many of these patients are symptomatic. Therefore, assuming all patients with a neurological co-infection have symptoms and there is only 1 case of true primary symptomatic CSF escape rather than secondary escape, the lowest possible estimate for the overall prevalence of primary symptomatic CSF escape is 1 in 1063, 0.09%, and the highest possible estimate is 27 in 1063, 2.5%. ** Perez Valero et al.; the prevalence of neurocognitive impairment in people with CSF escape was not significantly different to the prevalence of cognitive impairment in those without escape (38.2 vs. 37.7%; *p* = 0.91) [36]. *** Mukerji et al.; 22.2% of patients with CSF escape were categorised as having “Asymptomatic neurocognitive impairment”, defined as a deficit in neurocognitive testing that did not interfere with activities of daily living. These patients are included in the proportion of asymptomatic escape for the purpose of these prevalence estimates.

### 2.5. Mechanisms of CSF HIV RNA Escape

There are multiple possible mechanisms of CSF HIV RNA escape. One theory is reduced drug penetration of the CNS compartment, allowing for the evolution of drug resistance mutations within this compartment [53]. Another is that latent virus in the CNS reservoir reactivates. Once in the CNS, the virus infects and integrates proviral DNA into long-lived cells, such as microglia, that are relatively resistant to viral-induced apoptosis [63]. When activated, these cells maintain the CNS reservoir and can produce infectious virus [64,65]. Myeloid cells are intrinsically relatively resistant to productive infection due to the restriction factor sterile alpha motif domain and histidine–aspartic domain-containing protein 1 (SAMHD1) that blocks reverse transcription by depletion of deoxynucleotide triphosphate (dNTP) [66,67]. However, macrophage cell cycle transitioning can lead to the deactivation of SAMHD1 and render macrophages susceptible [68]. This transition can occur following various environmental cues, such as low oxygen. Myeloid reservoirs for HIV have become increasingly recognised as significant [69]. Other mechanisms include increased trafficking of T cells across the BBB in people with low-level viraemia (LLV) and clonal expansion of expanded infected T cells [65].

#### 2.5.1. Symptomatic Escape

Recent work by Kincer et al. has demonstrated that symptomatic CSF HIV RNA escape is driven by ongoing HIV replication in CNS CD4+ T cells [61]. In the study, there was concordance between the drug resistance mutations in the CSF and plasma virus, suggesting ongoing replication rather than compartmentalised infection [61]. The authors proposed a model whereby resistance to drugs with high CSF penetration, such as lamivudine, evolves during a period of treatment interruption and is selected on reinitiation of ART. Variants with lamivudine resistance due to the M184V mutation have reduced fitness and are increasingly susceptible to tenofovir, suggesting that they could only survive in an environment where tenofovir levels are not suppressive, such as the CSF [61]. This theory explains the increased prevalence of M184V mutations in people with CSF HIV RNA escape.

CSF HIV RNA escape can cause neurological symptoms by triggering CD8 encephalitis, a severe inflammatory disorder characterised by the infiltration of CD8 T-lymphocytes into the CNS. This rare but life-threatening disorder typically presents when HIV is well controlled on ART. The pathophysiology of CD8 encephalitis is unclear, but CSF HIV RNA escape is a common finding present in 68% of patients with CD8 encephalitis [70]. It has been suggested that ongoing and potentially compartmentalised CNS HIV replication induces a widespread CD8+ T cell response, CNS infiltration and encephalitis.

#### 2.5.2. Asymptomatic Escape

Asymptomatic CSF HIV RNA escape has been identified in patients who have had lumbar puncture for research purposes. Eden et al. found a CSF HIV RNA escape prevalence of 10% amongst 69 asymptomatic, virologically suppressed individuals [32]. A follow-up retrospective longitudinal study in a group in 75 asymptomatic individuals found a prevalence of 36% [71]. However, this is at a viral load limit of detection of 20 copies/mL compared with 50 copies/mL in the prior study. Upon repeat sampling, CSF HIV RNA escape persisted at a rate of 3%. It is thought that the other cases were due to CSF viral blips. In larger cohorts, including CHARTER, National NeuroAids Tissue Consortium (NNTC) and HIV Neurobehavioural Research Centre (HNRC), the prevalence of asymptomatic CSF HIV RNA escape amongst aviraemic individuals was between 2.7 and 4.7% (c.f. 0.09–2.5% symptomatic CSF HIV RNA escape) (Table 1) [35,36]. These were based on a limit of quantification of 50 copies/mL. It is likely that the true prevalence of asymptomatic CSF HIV RNA escape is greater than that of symptomatic CSF HIV RNA escape; however it is affected by the limit of detection of the assay used, the population from which the sample is drawn and the CSF sample volume used.

Asymptomatic CSF HIV RNA escape has been found to be associated with pleocytosis and biomarkers of intrathecal immune activation and BBB dysfunction, although not in all cases [32,57,72,73]. In contrast to symptomatic CSF HIV RNA escape, which involves active replication of HIV-1, asymptomatic CSF HIV RNA escape can be produced by virus expression from clonally expanded CD4+ T cells in the absence of replication or by replication in reactivated macrophages [65]. Eden et al., in a cohort of asymptomatic CSF HIV RNA escape, found raised neopterin, a marker of macrophage activation, which suggests that the source can be the CNS reservoir [32].

The concept of a plasma blip, an isolated detectable HIV RNA viral load followed by a return to viral suppression, is well recognised. Longitudinal analysis of neurologically asymptomatic and virally suppressed cohorts demonstrates that transient low-level detectable viraemia also occurs in the CSF [71]. This was not sustained in most patients and was associated with a plasma blip at one of the time points and a higher CSF neopterin [71]. This suggests that in well-controlled virologically suppressed patients, CSF viraemia may reflect low-level variations in the release of virus into the CSF compartment rather than ongoing replication within the CNS. The significance of asymptomatic CSF HIV RNA escape in terms of the impact on cognitive function or progression to symptomatic escape is currently unclear.

#### 2.5.3. Secondary CSF HIV RNA Escape

**Figure 1:**
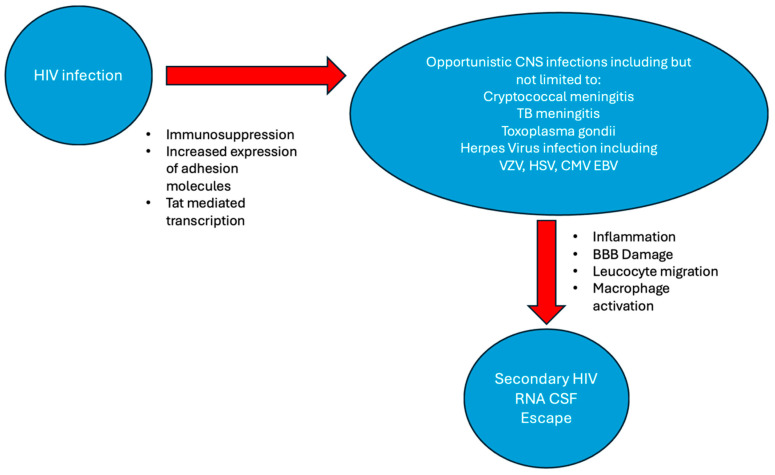
A schema to explain the mechanism of secondary CSF HIV RNA escape.

Figure 1 illustrates the mechanisms of secondary CSF HIV RNA escape. HIV can increase the risk of CNS opportunistic infections (OIs), including *Mycobacterium tuberculosis*, *Toxoplasma gondii*, herpes viruses, JC virus, Epstein-Barr virus (EBV) and *Cryptococcus neoformans*, by facilitating entry, enhancing transcription and causing reactivation [74]. In turn, opportunistic infections can generate inflammation and increase the translocation of HIV-infected lymphocytes across the BBB [74]. OIs, particularly those that cause macrophage activation such as Cryptococcus neoformans, tuberculosis and toxoplasmosis, can help to sustain local HIV replication in the CNS and the development of a distinct CNS virus [75,76]. Those with CNS OIs can have increased CSF HIV RNA viral loads and are at increased risk of escape, the significance of which is not known.

Persons with HIV are less likely to be able to completely control DNA viruses such as EBV. EBV is known to infect endothelial cells and, upon reactivation, increase the expression of proinflammatory cytokines CCL-2 and CCL-5 and adhesion molecules [77]. It is thought that this can lead to alteration of the BBB and that a chronic subclinical EBV infection could increase immune cell trafficking and thus the migration of HIV into the CNS [49,78]. A cohort study of 297 persons with HIV found that EBV was detected in the CSF of 9.2% of peripherally suppressed patients [79]. The presence of EBV was independently associated with CSF HIV RNA escape, CSF pleocytosis and markers of inflammation. A study using next-generation sequencing with immunostaining and multiplexed self-antigen serology demonstrated that EBV was more frequently present in the CSF of symptomatic CSF HIV RNA escape patients than in asymptomatic controls, suggesting that EBV may be playing a role in HABI [79].

#### 2.5.4. Artefactual—Differential Suppression and Limitations of the Assay

The pharmacodynamics of ART are such that in some cases, plasma viral load can suppress more quickly than CSF HIV RNA, particularly in those with HABI symptoms, low peripheral CD4 counts and reduced CSF pleocytosis [64,80,81]. It has been proposed that in some patients with advanced disease, T-tropic variants in the blood are rapidly suppressed, whereas M-tropic variants maintained in the CNS are suppressed more slowly [73]. Therefore, if the lumbar puncture is taken early in treatment, after treatment interruption and reinitiation or change in treatment, the CSF viral load may be greater than the plasma viral load, but this does not represent ongoing replication in the CNS. For example, in a recent CONNECT study, transient CSF escape was observed in a participant switching from efavirenz to dolutegravir, which was proposed to be due to differential viral decay between the CSF and plasma compartments [82]. Ellis et al. demonstrated that CSF viral decay following treatment initiation is much slower than the decrease in plasma viral load in people with advanced HIV disease (defined as a CD4 count <400/μL) and in those with cognitive impairment when compared with those who start treatment with a CD4 count >400/μL [80]. This suggests that in advanced HIV disease, there can be ongoing replication in the CNS compartment, which may take longer to respond to ART [80].

Particularly at low copy numbers, there is significant variation within and between HIV RNA assays. Intra-assay variability can be high and can be affected by many factors, including sample preparation, the operator’s experience and automaticity of the assay [50,83,84]. For example, at 50 copies/mL, precision has been estimated to be around 0.33 log10 or 23–110 copies/mL [85]. Given the sensitivity of novel tests, which can detect viral loads down to 20 copies/mL, a proportion of people will have CSF escape based on these results that would not have been classified as having escape with standard assays. For example, in Eden’s cohort, 27 participants (36%) were classified as having escape based on a limit of 20 copies/mL, whilst this reduced to 17 participants (20%) when the limit of 50 copies/mL was used [71]. The significance of this low-level CSF viral load is debated [86,87].

### 2.6. Biomarkers for Early Detection

Gisslén et al. showed that neurofilament light protein (NFL), a marker of axonal injury, was a useful biomarker for predicting the onset of neurocognitive impairment in people with HIV [88]. However, there are currently no biomarkers used in clinical practice to predict the onset of either symptomatic, asymptomatic or secondary CSF HIV RNA escape. NFL has the potential to be a useful marker for assessing treatment response. It is raised in symptomatic escape but normalises on CSF viral suppression; furthermore, as the plasma concentration correlates strongly with the CSF concentration, this has the potential to be a less invasive marker to monitor treatment response and to distinguish between legacy and active HIV-induced neuronal damage [60,89,90]. Hu et al. recently examined the role of over 1000 CSF proteins in HIV associated disease and found that for a panel of 10 proteins that were most useful in differentiating the different phenotypes of CSF HIV RNA escape, most of these proteins were involved in neuroinflammatory processes [60]. The role of these tools in detecting early CSF HIV RNA escape has yet to be established.

## 3. Existing Treatment for CSF HIV RNA Escape

Treatment for CSF HIV RNA escape is determined by the underlying cause and the symptomology. Secondary CSF HIV RNA escape is managed by treating the underlying cause. Treatment is currently only recommended for primary CSF HIV RNA escape when there are symptoms [30]. Therefore, the rest of this review will focus on the treatments for symptomatic CSF HIV RNA escape. Table 2 shows the strengths and limitations of each treatment, the current recommendations based on BHIVA and EACS guidelines and the level of evidence that supports the recommendation.

### 3.1. Optimising Antiretroviral Therapy

A potential issue in the treatment of HIV in the CNS may be the variability in the penetration of some antiretrovirals across the BBB to reach optimal concentrations in the CNS [35,91,92,93]. The BBB is a selective and highly impermeable barrier formed by a monolayer of microvascular endothelial cells connected by tight junction proteins [23]. The BBB serves an important purpose in the preservation of the microenvironment of the brain [23].

ART does not easily cross the BBB and, when it does, is susceptible to removal via ATP-binding efflux pumps embedded within the endothelial monolayer [94]. It is thought that some drugs, such as abacavir and protease inhibitors, are unable to reach therapeutic concentrations in the brain due to high efflux [94]. A multitude of factors contribute to the bioavailability of a drug in the CNS, including weight, molecular size, lipophilicity, interaction with molecular pumps and other proteins. For example, some drugs, such as the relatively new non-nucleoside reverse transcriptase inhibitor doravirine, reach very high unbound concentrations in the CSF and have been proposed as a potential useful addition in people with neurocognitive impairment [95].

There has been debate about the utility of a CNS penetration effectiveness (CPE) score to optimise ART in cognitive impairment. The CPE score ranks individual ART drugs from 1 to 4, with 4 reflecting the highest CNS penetration [96]. The score is derived from the chemical properties of the drug, the concentration of the drug in the CSF in human or animal studies and demonstrated effectiveness in improving CSF viral load in clinical studies [80,96,97,98]. The use of this score in clinical practice is limited, and cohort studies report conflicting findings. One small (*n* = 49) prospective randomised control trial found no difference in cognitive outcomes in those assigned to the higher CPE-score ART regimes and lower levels of plasma viral suppression [99]. There are some clear issues with the assumption that CSF drug levels are comparable with brain parenchymal levels; indeed, a recent study performed on the brains of recently deceased people with HIV found that for some drugs, such as efavirenz, parenchymal levels were much higher than CSF levels [100]. Therefore, using the CPE score to guide clinical practice is not recommended.

Whilst viral suppression is neuroprotective, there has recently been more recognition of the neurotoxicity of antiretroviral therapy [101]. Whilst some drugs, such as efavirenz, are well known to be neurotoxic, there are several questions about the potential toxicity of multiple ART drugs [102]. Treatment of patients with HABI and CSF HIV RNA escape needs to consider the potential neurotoxicity of the treatment itself.

### 3.2. Optimising ART to Target Resistant Viruses

Certain HIV drug resistance mutations, such as M184V/I mutations and thymidine analogue mutations, are more common in people with HIV and CSF HIV RNA escape [35,39]. This has informed the recommendation to adjust ART regimes based on resistance genotype [46,47]. Canestri et al. reported a case series in which CSF HIV RNA escape was present in 8/11 patients who had acute or subacute neurological symptoms [53]. Most (seven out of eight) of the patients with CSF HIV RNA escape had drug resistance mutations in the CSF virus. Following ART adjustment based on genotyping, all patients improved, and their CSF normalised [53]. This finding has been replicated in further retrospective case reviews and series and incorporated into international guidelines and is the standard of care [10,103].

### 3.3. Integrase Inhibitors Can Be Used to Intensify ART Regimens in CSF HIV RNA Escape

Henderson (2023) carried out a retrospective cohort study of 114 patients undergoing CSF analysis for clinical indications over a 5-year period between 2017 and 2022. Nineteen of these patients had CSF HIV RNA escape [55]. Of these 19 patients, only 17% (3 patients) were on an integrase inhibitor-based regime compared with 48% (36 patients) of those who did have CSF HIV RNA escape, and this was statistically significant, with *p* = 0.017. In contrast, over half (55%) of those with CSF HIV RNA escape were on a PI-based regimen compared with 36% of those who did not have CSF HIV RNA escape. In 10 of the 19 patients with CSF HIV RNA escape, the clinician thought escape was the cause of their neurological symptoms. In eight cases, an integrase inhibitor was added to the ART regimen (dolutegravir in seven cases and raltegravir in one case), and in three cases, boosted PIs were added (darunavir/ritonavir in two cases, and lopinavir/ritonavir in one case). Two participants had no change in their ART regimen. All 10 participants had symptomatic resolution. The numbers are small, but the optimisation of ART following resistance testing by adding integrase inhibitors may be useful in the management of CSF HIV RNA escape, given that currently, integrase inhibitor resistance is rare. Currently, the EACS guidelines recommends avoiding once daily dosing of raltegravir in CSF HIV RNA escape due to a lack of evidence in this cohort. A randomised open-label pilot study found that raltegravir intensification did not reduce CSF HIV viral load in people with symptomatic escape [104]. There is high inter-person variability in CSF concentrations and raltegravir, and there have been case reports of CSF raltegravir resistance [105,106]. Therefore, the use of raltegravir in CSF HIV RNA escape is not recommended.

### 3.4. Protease Inhibitors, Particularly Atazanavir, Are Associated with CSF HIV RNA Escape

In a large prospective study of 1063 participants on ART, Mukherji et al. examined the association between ART regimen and CSF HIV RNA escape [35]. In this study, with a median follow-up time of 4.4 years, 65% of patients were on a PI-based regimen, and CSF HIV RNA escape occurred in 7.2% of participants (*n* = 77). There was no significant difference in the CPE scores between those with and without CSF HIV RNA escape (*p* = 0.3). In adjusted analyses, regimens containing protease inhibitors were independent predictors of CSF HIV RNA escape (OR 3.1, (95% CI 1.8–5.0)), with regimens containing atazanavir being most associated with escape compared with non-ATV + nucleoside reverse transcriptase inhibitor (NRTI) regimens (OR 3.1 95% CI 1.9–5.3). In a cohort of 513 people with HIV in India, symptomatic CSF HIV RNA escape was present in 10.5% of the cohort; the most common ART regimen was tenofovir disoproxil (TDF)/emtricitabine (FTC) and atazanavir/r [107]. Participants either underwent intensification with the addition of zidovudine (AZT) (23 cases) or replacement of the boosted protease inhibitor (PI) (mostly AZT) with a more CNS penetrating PI such as lopinavir/ritonavir or darunavir/ritonavir plus the addition of an integrase inhibitor (9 cases); the third option was replacing the PI with a better CNS penetrating PI plus addition of AZT/3TC and TDF (4 cases). Follow-up CSF viral loads were suppressed in 18/24 patients who underwent PI/INSTI intensification and 15/23 patients in the AZT intensification group. All patients who had drug resistance mutation testing had viruses with the M184V mutation in their CSF.

### 3.5. CCR5 Inhibitors

Maraviroc inhibits entry of HIV-1 by binding to and modifying the CCR5 extracellular loops’ conformation so that they cannot bind to gp120. CCR5 is expressed on neurons, macrophages, microglia and glial cells. It plays a role in brain development and neuroimmunology [108]. It is thought that HIV infection can disrupt normal synaptic functions by altering cytokine and chemokine levels, activating aberrant intracellular signalling and leading to neuroinflammation and neuronal death [109]. Neuronal activation via numerous CD4-independent pathways, including p38-MAPK, neuronal nicotinic receptor α7 and NMDA receptors, are shown to lead to increased calcium ion influx and apoptosis [108].

Maraviroc penetrates the BBB well and achieves concentrations well above the concentration required to inhibit viral replication by 90% (IC90) in the CSF [110,111,112]. As previously discussed, viruses within the CNS are more likely to be macrophage-tropic and utilise the CCR5 co-receptor [28,113,114]. In macaques, maraviroc monotherapy has been shown to reduce both replicating and latent SIV in the brain [115]. One study of six patients with symptomatic CSF HIV RNA escape found significant improvement in CSF viral load one month after starting maraviroc [111]. Whilst pilot studies have shown a signal towards improvement in neurocognitive performance with maraviroc [116,117], a larger (*n* = 49) randomised placebo-controlled trial showed no effect [118].

CCR2+ monocytes are thought to be associated with the degree of cognitive impairment [119,120]. This is thought to be due to the preferential trafficking of these cells into the brain, contributing to neuroinflammation and reduced cognitive function [119,121,122]. An open-label trial of a CCR2 and CCR5 inhibitor cenicriviroc (*n* = 17) showed some improvement in cognitive impairment and decreased levels of biomarkers associated with inflammation (sCD163, sCD14 and neopterin); the study did not explore the effect on CSF viral load [123].

### 3.6. Two Drug Regimens and Long-Acting Antivirals

It is recommended to avoid two drug regimens in people with CSF HIV RNA escape. This recommendation is based on data from a Swiss cohort of 25 people with CSF HIV RNA escape and 263 without, in which there was a slightly higher proportion of participants on two drug regimens including dolutegravir and rilpirivine or lamivudine in the group with CSF HIV RNA escape compared with those without (4.0% vs. 1.5%, *p* = 0.07) [43].

At present, there is no recommendation on the use of long acting antiretrovirals in cases of CSF HIV RNA escape. Initial data suggest that the long-acting injectable drugs cabotegravir and rilpirivine reach good concentrations in the CSF [124]; but there is insufficient data on the use of long-acting ART in cases of known CSF HIV RNA escape. Long-acting ART may be a useful tool in managing patients with poor ART adherence due to cognitive impairment [125].

### 3.7. Preventing CSF HIV RNA Escape Before It Occurs

Whilst the bulk of this review focuses on the treatment of CSF HIV RNA escape, it is crucial to recognise that escape is more likely to occur in those who have had disruptions in ART, multiple different drug regimens and resistance. Therefore, an important step in preventing escape is optimising the ART regimen and adherence to avoid the development of resistance.

## 4. Repurposing Licensed Drugs for CSF HIV RNA Escape

### 4.1. Lithium

Early studies in murine models of HIV showed that lithium reduced the development of lymphadenopathy, splenomegaly and lymphoma associated with immunodeficiency; lithium also increased survival [126]. Four small-scale trials have looked at the utility of lithium in treating cognitive impairment defined using the HAND criteria [127,128,129,130]. The results of these trials are mixed; all trials report that lithium was safe and well tolerated, but the evidence of an effect on neurocognitive outcomes or CNS biomarkers of neuroinflammation was minimal [27,107]. Only one of these studies looked directly at the impact on CSF viral load and found that whilst there was a significant improvement in neuropsychological (NP) performance following treatment with 12 weeks of oral lithium at a dose of 300 mg per day, there was no effect on plasma or CSF viral load from baseline. This suggests that the effect of lithium on NP performance in these participants was independent of CNS viral replication. This study was limited in size (*n* = 8), duration and lack of a control group [127].

### 4.2. Valproic Acid

Sodium valproate and lithium are known to inhibit glycogen synthase kinase-3 beta (GSK3B), a serine/threonine kinase upregulated by the HIV proteins Tat and gp120. GSK3B inhibitors, such as valproate, have been shown in vitro to reduce HIV-1-mediated neurotoxicity, though this has yet to be demonstrated in vivo [131].

Valproate acts as a histone deacetylase (HDAC) inhibitor. HDAC is a chromatin remodelling enzyme that maintains latency of integrated HIV provirus; inhibiting this enzyme leads to re-activation of the virus and can be used in shock and kill strategies. A proof-of-concept study in four volunteers by Lehrman et al. (2005) used valproate with enfuvirtide and demonstrated a potential acceleration in the clearance of HIV from resting T cells [132]. However, larger studies have not replicated these results [133,134].

Whilst a small-scale pilot study suggested a trend towards cognitive improvement in people with HIV treated with valproate as an adjunctive therapy, the opposite was the case in a larger clinical observational study using higher doses of valproate (850 mg/day) and with a longer follow-up period [135,136]. Retrospective cohort reviews, although small in size, have shown no evidence that valproate affects CSF viral load when given for 12 weeks [137]. Given the potential neurotoxicity of valproate and the efficacy of ART optimisation for symptomatic CSF HIV RNA escape, it is not recommended.

### 4.3. Statins

HMG Co-A reductase inhibitors have both beneficial cardiovascular and immunomodulatory effects. There is some in vitro work suggesting that statins may inhibit HIV replication by reducing membrane lipid rafts that are rich in chemokine receptors, reducing adhesion molecule expression and reducing Rho guanosine triphosphatase activity [138,139,140,141].

In one large cross-sectional study, Letendre et al. (2007) analysed the impact of statin use on HIV viral load in the CSF [142]. A minority, 10%, of the 658 people with HIV in the CHARTER cohort who had baseline NP testing, plasma and CSF HIV viral load testing were using a statin. Amongst those taking ART, participants taking a statin were less likely to have a detectable CSF viral load (OR = 0.09, *p* < 0.001). However, statin use was not significantly associated with CSF viral load in the multivariate logistic regression analysis adjusted for AIDS diagnosis, ART use, CD4 count, HIV RNA plasma level, depression scores, education, age, gender and ethnicity. This cohort study has the benefit of relatively large numbers but is limited by the cross-sectional design and pretreatment difference between the groups; furthermore, the dose and class of statin varied between participants with five different drugs used, each of which had different lipophilicity and doses not reported. The power of this study to detect associations is limited, but given the trend towards significance, further research is warranted.

### 4.4. Selective Serotonin Reuptake Inhibitors

In the CHARTER cohort, 195/658 (30%) of the participants were taking selective serotonin reuptake inhibitors (SSRIs) [142]. Selective serotonin reuptake inhibitors were associated with a lower risk of having detectable HIV RNA in the CSF (odds ratio: 0.69, *p* = 0.05) [142]. The authors combined those taking three drugs—citalopram (*n* = 24), trazodone (*n* = 64) and sertraline (*n* = 37)—that were trending towards significance into one category, the so-called “antiviral serotine reuptake inhibitors” and found that there was greater protection from detectable HIV RNA in the CSF (OR: 0.56, *p* = 0.02). This effect was more significant in those not taking ART; the authors, therefore, concluded that this effect cannot be attributed to improved mood and ART adherence when taking SSRIs.

Those using both an SSRI and a statin had a lower proportion of detectable HIV RNA in the CSF compared with those taking either of the drugs individually or neither. The authors comment that ART use was similar between the two groups, but given the low numbers and multiple potential confounders, it is difficult to know whether this is a true reflection of the therapeutic effect. This analysis is limited by the potential for multiple confounders, the grouping of many different drugs and the artificial grouping of proposed antiviral SSRIs. The analysis does not separate those with and without peripheral viral suppression, which makes it difficult to ascertain whether SSRIs would be genuinely useful in those with CSF HIV RNA escape. There is little understanding of a potential mechanism by which SSRIs would reduce HIV replication, and further work is required [142,143].

### 4.5. Minocycline

Minocycline, a tetracycline antibiotic, has known immunomodulatory effects, and in animal models of CNS HIV, minocycline has been demonstrated to reduce lentiviral infection in microglia [144,145,146,147]. There is in vitro evidence that minocycline has antiviral effects on human CD4+ T cells in the context of HIV infection [148]. One open-labelled pilot study in humans tests the hypothesis that minocycline will reduce HIV CNS viral load both absolutely and in relation to plasma viral load [149]. Seven participants, none of whom were taking ART, were included in the final analysis. There was no change in the CSF or plasma HIV-1 RNA level [149].

### 4.6. Janus Kinase (Jak 1/2) Inhibitors

Ruxolitinib has been investigated in preclinical and early clinical studies and was found to reduce markers of immune activation and the peripheral HIV reservoir [150]. Baricitinib has a favourable safety profile and reaches therapeutic CNS concentrations in humans. It has shown promise in murine models, non-human primates and humans [151,152]. It is currently undergoing evaluation in Phase II clinical trial therapy to determine if it decreases the HIV CNS reservoir in PLWH with durable virologic suppression on ART.

## 5. Novel Therapeutics for Treating CSF HIV RNA Escape

### 5.1. The Use of Nanoparticles to Cross the BBB

We have discussed the optimisation of CNS penetrance of antiretroviral therapy. However, this is not the magic bullet for CSF HIV RNA escape. Taking ART which penetrates the CNS is associated with neurovascular toxicity [153,154,155]. If adequate concentrations are not reached, then clonal expansion of these HIV reservoirs will continue. This clonal expansion allows for the development of multidrug-resistant strains of HIV, which are more difficult to target with conventional ART regimens. Any treatment interruption leads to the expansion of this viral reservoir and rebound viraemia [156,157].

Nanotechnology has been proposed as a solution to design drugs that can cross the BBB whilst preserving its integrity [158]. Nanoparticles are materials with overall dimensions less than 100 nm; those designed for brain specific drugs are typically administered intranasally for direct delivery [159]. There has been some progress in using nanotechnology to achieve a nanoparticle delivery of conventional ART drugs to solve the problems associated with delivering ART across the BBB [160,161]. Nanoparticles can be adapted with surface moieties to increase specificity to desired cells. One study used a poloxamer-PGLA-based nanocarrier for elvitegravir (an integrase inhibitor) and showed that, in vitro, this carrier crossed the BBB and achieved viral suppression in HIV-1 infected macrophages [162]. Another in vitro model utilised surface modified nanodiamonds to deliver efavirenz across the BBB [163]. This technology has also been used in rodent models to demonstrate improvement in macrophage-mediated uptake of ART [164,165]. There are currently no clinical trials in nanomedicine for the treatment of HIV, but this remains an exciting future perspective for both better treatment of CNS disease and elimination of the HIV reservoir [166].

### 5.2. Elimination of the CNS Reservoir

We have discussed a few approaches to achieve reduced viral replication in the CNS. One approach discussed by Nühn et al. in their comprehensive 2022 review is that of “shock and kill” [167]. This approach aims to reactivate the latent reservoir with latency reversal agents, including epigenetic modifiers, intracellular signalling modulators, cytokine or immune receptor agonists and transcription elongation factors, which will then be eliminated by the immune system or by viral cytolysis, achieving viral cure. Very few latency reversal agents have been studied in vivo, but it is thought that some degree of immune stimulation will be required to stimulate clearance [168]. The CNS reservoir, likely composed of microglia, astrocytes and CD4+ T cells, provides a unique challenge. There are pharmacokinetic challenges as LRAs need to be able to cross the blood–brain barrier; but more importantly, we need to consider whether viral reactivation within the CNS is a tolerable side effect. One in vivo study in a macaque SIV model showed that the administration of ingenol-B and vorinostat led to a reactivation of the CNS reservoir, but there were also signs of increased neuronal degradation, inflammation and encephalitis [169]. The neuronal damage provoked by HIV reactivation is unlikely to be a tolerable side effect in humans.

There are also specific challenges in the “kill” stage in the CNS. As discussed, myeloid cells that make up the CNS reservoir are more resistant to virus-induced cytopathy, and viral infection further upregulates gene expression associated with apoptosis-resistance [170]. CD8+ T cell-mediated killing of infected macrophages requires high concentrations of interferon gamma and long cell-to-cell contact times [171,172]. In vitro work suggests a level of resistance to virus-induced apoptosis in primary CNS cells [173,174]. Immune-mediated clearance of infected cells in the CNS is unlikely to occur without causing inflammation and encephalitis and neuronal damage [175]. This is unlikely to be an acceptable cost for an unproven benefit.

**Table 2:** Summaries of the treatment strategies for CSF HIV RNA escape, their advantages and limitations and the strength of evidence and recommendations based on current guidelines.

Treatment	Advantages	Limitations	Strength of Evidence	Recommendation (Strength of Recommendation)
ART optimisation based on resistance profiles	Tailored regimen for each patient	Resource intensive Access to genotyping is limited in low-resource settings	Low Case series [39,53,61,107]	Recommended (Strong)
ART optimisation by adding a second generationintegrase strand inhibitor (INSTI)	Cost effective; easily available Second generation INSTI’s now recommended in most first line regimes Guidelines suggest doubling dose of DTG if concerned re INSTI resistance	Neurocognitive side effects with dolutegravir in some patients Raltegravir should be avoided in suspected CSF escape	Low Case series [43,107] Expert opinion	Recommended (Weak)
ART optimisation based on CNS penetration effectiveness (CPE) scores	Cost effective as does not require resistance testing.	Relies on the assumption that CSF drug levels are comparable with parenchymal levels. Not backed up by evidence from autopsy studies showing much higher levels in parenchyma than CSF [102]. Need to consider the potential neurotoxicity of ART itself [104]	Low Case series and retrospective cohort studies [176,177,178,179].	Not recommended
ART optimisation—avoiding PI based regimens	Cost effective	PI-based regimes are the only option for second-line treatment in some contexts.	Low A large retrospective cohort study (n = 1063) [35].	Recommended (Strong)
ART optimisation—adding CCR5 inhibitors	Good BBB penetration	No effect in larger randomised controlled trials	Low Case series [114] One randomised controlled trial (*n* = 49) [118]	Not Recommended
ART optimisation—avoiding dual ART regimens	Cost effective	Dual ART regimens are a simpler option for those with cognitive impairment	Low Retrospective cohort [43]	Recommended (Strong)
Lithium	Already licensed Safe and well tolerated	Risk of neurotoxic side effects, difficult to dose.	Low Pilot studies, no control group [127]	Not recommended
Valproic acid	Already licensed	Neurotoxic side effects Teratogenic	Low Retrospective cohort studies [137]	Not recommended
Statins	Easily available Cost effective Other cardiovascular and immunomodulatory benefits	Evidence from cross-sectional studies only. Pretreatment difference between the groups. Dose and class of statin varied in CHARTER.	Low Retrospective cohort studies [142]	Not recommended
Selective serotonin reuptake inhibitors	Cheap and easily available	Evidence from cross-sectional studies only. Multiple different SSRIs grouped together. Not all patients in CHARTER on ART; not necessarily generalisable to this population.	Low Retrospective cohort studies [142]	Not recommended
Minocycline	Cheap Already licensed	Only evidence from a small, open-labelled, pilot study No participants taking ART—not applicable to current population.	Low Open-labelled pilot study [147]	Not recommended
Baricitinib	Licensed	Currently in Phase II clinical studies	Preclinical studies in rodents and non-human primates [151,152]	Not recommended
Nanoparticles	Optimise concentration in CNS Could both treat CSF escape and help with elimination of the reservoir	Not yet in clinical trials	Insufficient in vitro and rodent models [162,163,164,165,166]	Not recommended
Elimination of the CNS reservoir with “shock and kill” strategies	Elimination of the CNS reservoir essential to achieve HIV cure	Neuronal damage provoked by HIV reactivation and immune mediated clearance is unlikely to be a tolerable side effect in humans	Insufficient In vitro and rodent models [167]	Not recommended

Strong recommendations are shown in green, weak recommendations in orange and interventions that are not recommended are shown in red.

## 6. Conclusions

Whilst ongoing HIV replication in the CSF is not the only cause of cognitive impairment in people living with HIV, it is an important factor and is known to contribute to HABI. CSF HIV RNA escape can be difficult to manage and can recur following mild infection or change in antiretroviral treatment [180]. Antiretroviral therapy optimisation to target resistant virus is the only recommended strategy for treatment, though the evidence for this comes from relatively small studies. There has been some exploration into licensed drugs to explore their effect on CNS disease; the evidence is limited to poorly controlled pilot studies and case series. Given the evidence of immune activation in CSF HIV RNA escape, ongoing Phase II clinical trials utilising Janus kinase (Jak 1/2) inhibitors to decrease the HIV CNS reservoir and immune activation are promising. Newer treatments using nanotechnology to facilitate crossing the BBB have the potential to change the management of CSF HIV RNA escape but have yet to be studied in clinical trials.

Uncertainly remains around the implications of asymptomatic CSF HIV RNA escape and its clinical significance. Longitudinal follow up cohorts with asymptomatic CSF HIV RNA escape will be invaluable in clarifying their clinical outcomes. In addition, research on the dynamics of biomarkers of CNS immune activation and neuroinflammation in this cohort is required. It is important to note that very few studies have examined CSF HIV RNA escape in sub-Saharan Africa, where the burden of HIV is greatest. Therefore, research into the prevalence and clinical spectrum is warranted in this setting.

The current recommended approach for managing CSF HIV RNA escape in symptomatic PLWH involves sequencing the CSF virus, targeting ART to overcome resistance and intensifying the ART regimen. It is also important to recognise that whilst whole genome sequencing for HIV is widely available in high-resource settings, access to HIV viral loads and genotypic resistance testing is limited in low-resource setting; this limits the ability to both diagnose and treat CSF HIV RNA escape. In recent years, the World Health Organization has worked to increase access to HIV drug resistance testing by increasing laboratory capacity. Point mutation assays have been developed for key mutations, which allow for more cost-effective access [181,182]. However, the cost effectiveness of this approach versus the intensification of ART regimens (for example, commencing twice daily dolutegravir in the context of low-population INSTI resistance) needs to be evaluated. If the BHIVA and EACS recommendation that all patients with suppressed viral load and new cognitive impairment should have their CSF taken for HIV viral load and resistance testing is to be deployed globally, it is essential that the logistical and financial barriers in implementing this recommendation in low-resource settings are addressed.

## Data Availability

No new data were created or analyzed in this study. Data sharing is not applicable to this paper.
